# Malignancy Presenting As Recurrent Empyema: A Report of Two Cases and a Systematic Review

**DOI:** 10.7759/cureus.109930

**Published:** 2026-05-30

**Authors:** Tommy Dharmawan, Muhammad Fahd Abdurrahman, Yandrevz Reminiscere Habasaron Silaban, Shakira Amirah

**Affiliations:** 1 Cardiothoracic and Vascular Surgery, Universitas Indonesia, Depok, IDN; 2 Cardiothoracic Surgery, Universitas Indonesia, Depok, IDN; 3 Emergency Medicine, Universitas Indonesia, Depok, IDN; 4 Obstetrics and Gynaecology, Universitas Indonesia, Depok, IDN

**Keywords:** diagnosis, empyema, infection, malignancy, tuberculosis

## Abstract

Lung malignancy presenting as empyema is a rare presentation, which is commonly associated with infection. We report two patients initially diagnosed with tuberculosis-associated pleural disease who were subsequently confirmed to have lung malignancy. The first case involved a woman in her 40s presenting with progressive dyspnea, hydropneumothorax, persistent exudative pleural drainage, and prolonged air leak despite anti-tuberculosis therapy. Repeated cytological and microbiological examinations were negative, while persistently low pleural adenosine deaminase (ADA) levels and lack of clinical improvement prompted thoracotomy biopsy, which confirmed adenocarcinoma. The second case involved a man in his 40s presenting with persistent empyema during tuberculosis treatment. Despite thoracotomy and decortication, the patient continued to develop progressive respiratory and systemic symptoms. Subsequent imaging demonstrated a chest wall mass and vertebral lytic lesions suggestive of metastatic disease, while histopathological examination confirmed squamous cell carcinoma.

A systematic search of PubMed, Cochrane, ProQuest, and Wiley databases up to May 5, 2026, identified eight published cases of lung malignancy presenting as empyema. Clinical presentation was frequently non-specific, although several patients exhibited red flag features such as hemoptysis, weight loss, and anorexia. Laboratory findings were not sufficiently specific to distinguish malignancy-associated empyema from infectious etiologies. Computed tomography frequently revealed findings suggestive of underlying malignancy, including lung masses, mediastinal lymphadenopathy, bronchial obstruction, and invasion into adjacent structures. Initial investigations commonly supported an infectious diagnosis, while a definitive diagnosis was consistently established only after invasive tissue biopsy.

Malignancy-associated empyema represents an important diagnostic pitfall, particularly in tuberculosis (TB)-endemic settings where infectious etiologies are strongly favored. Persistent or non-resolving empyema with atypical clinical, biochemical, or radiological findings should prompt early tissue biopsy to avoid delayed oncologic diagnosis and treatment.

## Introduction

Empyema remains a significant pleural infection, affecting approximately 80,000 patients annually in the United States and the United Kingdom, with reported 30-day mortality rates reaching 10.5% and exceeding 19% at one year [1]. Beyond its clinical burden, thoracic empyema also imposes substantial economic costs, with annual healthcare expenditures in the United States estimated to approach 500 million USD, largely driven by prolonged hospitalization, antimicrobial therapy, pleural drainage, and surgical interventions [1].

Clinically, empyema commonly presents with non-specific respiratory symptoms and is most frequently associated with infectious etiologies, often leading to an initial diagnosis of parapneumonic effusion [1]. In tuberculosis (TB)-endemic regions, pleural TB represents an additional and diagnostically dominant consideration [2]. While this infectious framework is epidemiologically justified, it may obscure less common underlying conditions. Malignancy presenting as empyema is rare, with reported incidence ranging from 0.1% to 0.3% [1], yet remains an important diagnostic challenge.

The diagnosis of malignancy-associated empyema is particularly difficult because its clinical, laboratory, and radiological findings frequently overlap with infectious pleural disease. Pleural fluid cytology may be negative for malignant cells, while imaging findings can be masked by inflammatory changes or loculated pleural collections, often delaying definitive diagnosis until invasive tissue biopsy is performed [3-10]. Consequently, malignancy-associated empyema remains under-recognized in clinical practice, particularly in TB-endemic settings. In this study, we present two cases of empyema in which the underlying diagnosis of malignancy was subsequently confirmed.

## Case presentation

Case 1

A woman in her 40s presented to the pulmonology outpatient clinic in June 2025 with worsening shortness of breath over the past week, accompanied by occasional episodes of hemoptysis. There were no constitutional symptoms, such as weight loss and sweating at night. The patient had a history of hypertension. No history of smoking. One month prior, the patient had been admitted to another hospital, where she underwent therapeutic thoracentesis for a large left-sided pleural effusion, yielding approximately four liters of fluid. Pleural fluid analysis at that time was suggestive of tuberculous pleural disease, and based on this clinical impression, the patient was commenced on standard anti-TB therapy.

Despite ongoing therapy, the patient reported progressive dyspnea, leading to referral to our institution for further evaluation. Initial chest radiograph revealed a left-sided hydropneumothorax with rightward mediastinal shift, as shown in Figure 1. A water-sealed drainage (WSD) system was placed the following day, and the pleural fluid analysis revealed a high-lactate dehydrogenase (LDH) exudate effusion based on Light’s criteria (pleural fluid-to-serum LDH ratio >0.6), with a pleural fluid-to-serum LDH ratio of 2.7 (424/157 U/L). The patient was administered intravenous ceftriaxone 2 grams per day. Histopathological examination of the hydropneumothorax pleural fluid specimen revealed no malignant tumor cells. Laboratory investigations revealed a significant leukocytosis, with a white blood cell (WBC) count of 12.51x10^3^/μL. The detailed results of the laboratory can be seen in Table 1. This significant elevation, combined with the radiologic evidence of a left hydropneumothorax and ipsilateral lung consolidation, strongly suggests a diagnosis of thoracic empyema.

**Figure 1:**
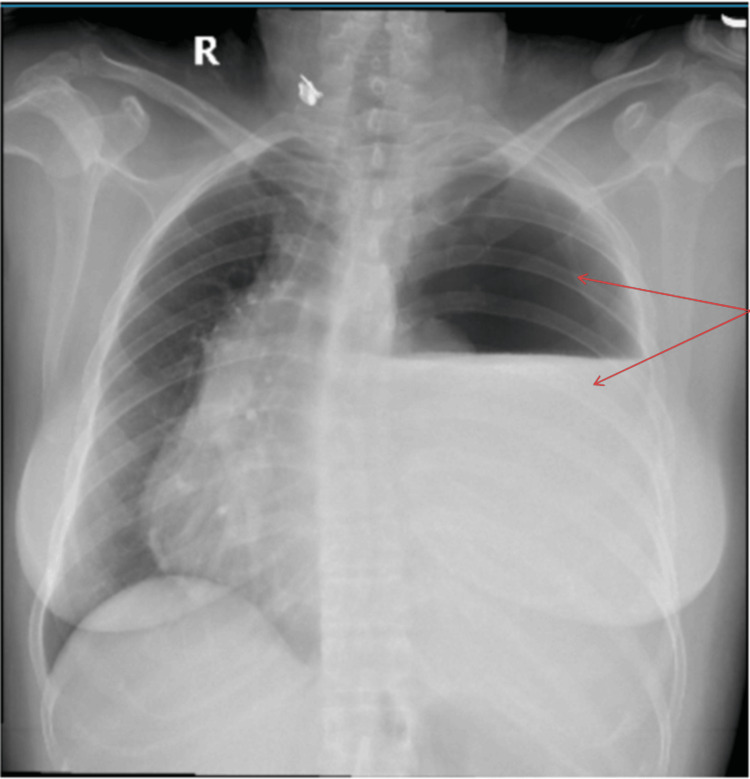
Case 1: Chest radiograph revealing a left-sided hydropneumothorax (red arrow) with rightward mediastinal shift.

**Table 1:** Laboratory results. CEA: carcinoembryonic antigen; ADA: adenosine deaminase; CA-125: cancer antigen 125; TB: tuberculosis

Test name	Result	Reference value	Unit
Leucocyte	12.51	4.00-10.00	10^3^/µL
CEA	21.54	<3.8	ng/mL
ADA	5	>40: TB <40: other than TB	U/L
CA-125	66.82	0.0-35.0	U/mL

High-resolution contrast-enhanced CT scan of the thorax, which was performed two days after the initial chest X-ray, demonstrated left hydropneumothorax with partial consolidation and ground-glass opacities in segments 1/2 and 10 of the left lung, as well as a “tree-in-bud” appearance in segment 4 of the right lung, as shown in Figure 2. No mediastinal or hilar lymphadenopathy was identified.

**Figure 2:**
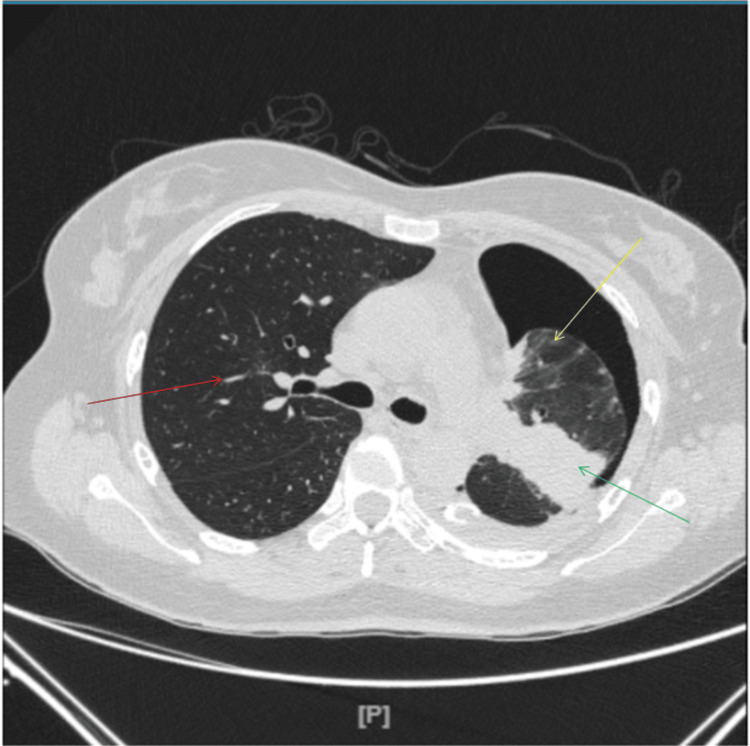
Case 1: CT scan of the thorax demonstrating left hydropneumothorax with partial consolidation (green arrow) and ground-glass opacities (yellow arrow) in segments 1/2 and 10 of the left lung, as well as a “tree-in-bud” appearance (red arrow) in segment 4 of the right lung. No mediastinal or hilar lymphadenopathy was identified.

After 10 days of hospitalization, the WSD system continued to demonstrate persistent bubbling with serous pleural fluid production of approximately 300-500 mL every 12 hours, suggesting prolonged pleural effusion accompanied by persistent air leak. Due to the lack of clinical improvement, the patient was scheduled for bronchoscopy with bronchial toilet and methylene blue evaluation for suspected fistula, followed by thoracotomy biopsy and pleurodesis.

Bronchoscopic evaluation did not identify a bronchopleural fistula, as shown in Figure 3. Acid-fast bacilli (AFB) examination and cytological analysis of bronchial fluid were also performed, both yielding negative results for acid-fast organisms and malignant cells. Subsequently, thoracotomy biopsy and pleurodesis were performed, and a new left-sided chest tube was inserted. Postoperative chest radiography findings are shown in Figure 4.

**Figure 3:**
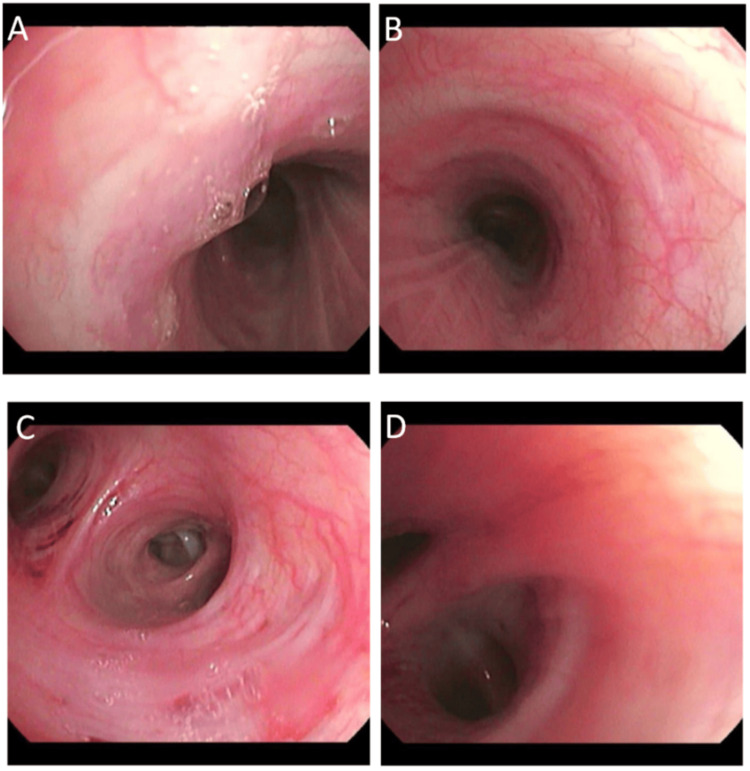
Case 1: (A) Left main bronchi, (B) left upper lobe, (C) lingula, and (D) left lower lobe demonstrating patent orifices with normal mucosa, without visible secretions or endobronchial abnormalities.

**Figure 4:**
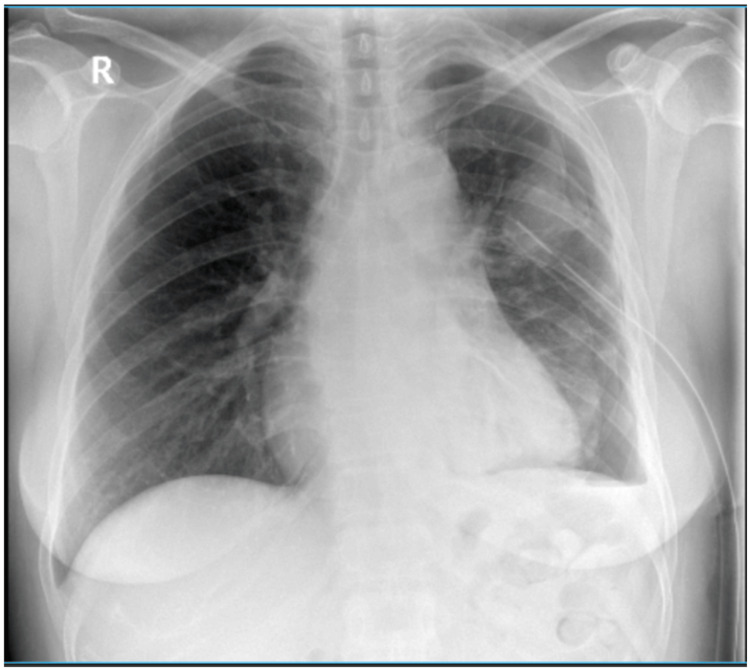
Case 1: Postoperative radiograph demonstrating decreased left-sided hydropneumothorax compared with the first radiograph, with persistent heterogeneous opacities in the upper and lower zones of the left lung.

Further laboratory evaluation demonstrated low pleural adenosine deaminase (ADA) levels of 5 U/L, elevated carcinoembryonic antigen (CEA) levels of 21.54 ng/mL, and cancer antigen 125 (CA-125) levels of 66.82 U/mL. Gynecologic consultation was obtained to evaluate the elevated CA-125 level. However, no gynecological malignancy was identified.

One week after thoracotomy biopsy and pleurodesis, the patient was discharged with the chest tube in place and continued anti-TB therapy. Two weeks later, during outpatient follow-up, the chest tube continued to produce 300-400 mL of serous fluid per 24 hours without further bubbling. An indwelling pleural catheter was subsequently inserted. At that time, histopathological examination results had become available and confirmed adenocarcinoma, as shown in Figure 5. The patient completed the sixth cycle of first-line platinum-based doublet chemotherapy, receiving a combination of pemetrexed and carboplatin for adenocarcinoma.

**Figure 5:**
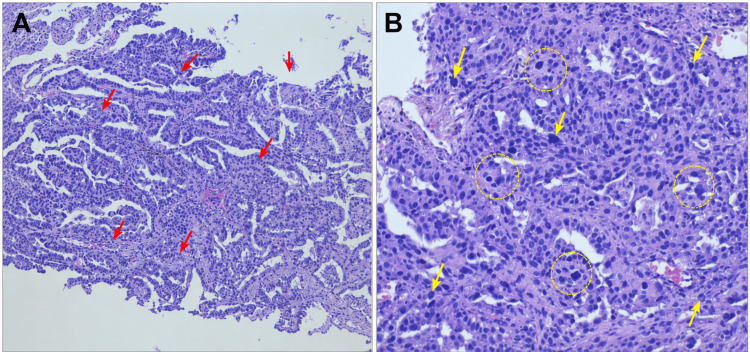
Case 1: (A) Tissue sections revealing a malignant tumor composed of infiltrative glandular and papillary structures (red arrow). (B) The tumor cells exhibiting pleomorphic, hyperchromatic nuclei (yellow circle) with eosinophilic cytoplasm (yellow arrow).

Case 2

A male in his 40s presented to the emergency unit in August 2023 with shortness of breath over the past week. The dyspnea worsened when lying supine, accompanied by pleuritic chest pain. The patient had a history of clinical lung TB and had already started the anti-TB drugs in June 2023. The patient came with a diagnosis of thoracic empyema due to TB in the third month on WSD.

On physical examination of the lungs, vesicular breath sounds were decreased on the right side, and a radiograph showed pleural effusion on the right side of the lung with a component of pneumothorax foci, right lung consolidation with no infiltrate or nodule in the left side of the lung, as shown in Figure 6. A thoracotomy and decortication operation was done in August 2023. Intravenous ceftriaxone 2 grams per day was administered to the patient. Analysis of the pleural fluid showed no growth of bacteria. Histopathological examination was not obtained during the initial procedure. The post-operation radiograph is shown in Figure 7.

**Figure 6:**
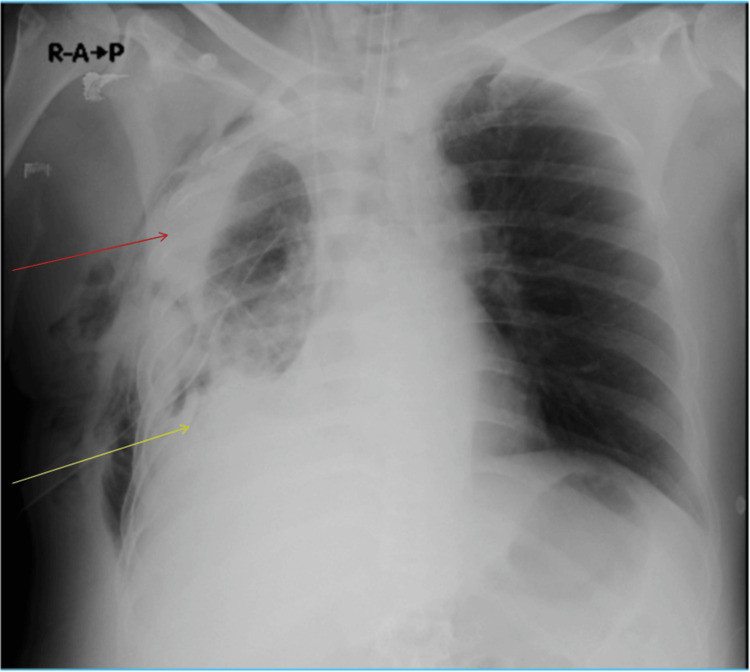
Case 2: Initial chest X-ray demonstrating a pleural effusion (yellow arrow) and a consolidation (red arrow) on the right side of the lung and a chest tube with the tip positioned at the level of posterior sixth rib.

**Figure 7:**
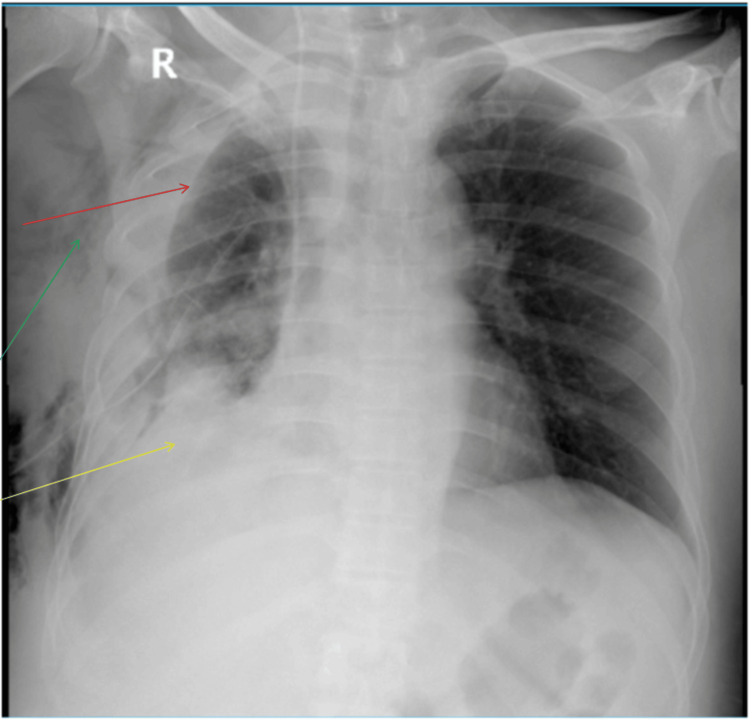
Case 2: Post-operation chest X-ray demonstrating right pleural effusion (yellow arrow) with focal pneumothorax components (red arrow) and right lung consolidation. Right lateral chest wall soft tissue swelling and subcutaneous emphysema extending to the right axilla (green arrow) were also noted. Endotracheal tube, central venous catheter, and right-sided chest tube were in appropriate position.

The patient was sent home in August with minimal symptoms and continued the anti-TB drugs. While continuing the TB medication, the patient complained of nausea, cough, dyspnea, and lower back pain. At the sixth month of medication, the patient underwent a thoracolumbar radiograph and CT scan for evaluation.

The result in Figure 8 shows a right-sided pleural effusion and a mass at the right fifth intercostal muscle from a CT scan and a lytic lesion with destruction at the T12th vertebral corpus from a thoracolumbar radiograph suggestive of a bone metastasis. The development of new symptoms and the identification of a chest wall mass and lytic lesions prompted further evaluation for malignancy. The patient underwent CT-guided transthoracic needle aspiration (TTNA) and core biopsy. The pleural fluid sample from TTNA was subjected to histopathological analysis, which revealed squamous cell carcinoma, as shown in Figure 9. The patient was then referred to another hospital for the treatment of squamous cell carcinoma.

**Figure 8:**
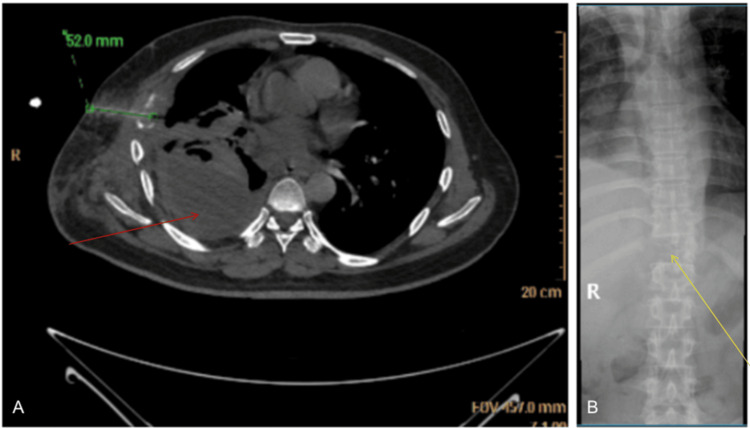
Case 2: (A) CT scan-guided transthoracic biopsy showed a right-sided pleural effusion and a mass at the fifth right intercostal muscle (red arrow). (B) The thoracolumbar radiograph showed a lytic lesion at the T12th vertebral corpus (yellow arrow) suggestive of a bone metastasis.

**Figure 9:**
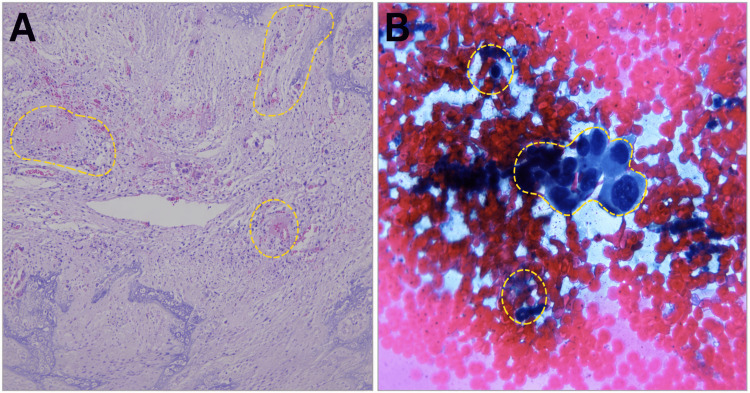
Case 2: (A) Infiltrative clusters of tumor cells with pleomorphic nuclei (yellow circle) were identified within bone, cartilage, and connective tissue. (B) The tumor cells exhibited pleomorphic, hyperchromatic nuclei with abundant dense cytoplasm (yellow circle).

## Discussion

Empyema is paradigmatically framed as an infectious disease, and in most clinical settings, this framing is epidemiologically defensible. Approximately 20% of patients with bacterial pneumonia will develop a parapneumonic effusion, a proportion that rises substantially in immunocompromised hosts [1]. In TB-endemic regions such as Indonesia, where more than one million incident cases are recorded annually, pleural TB constitutes an additional and diagnostically dominant etiology [2]. The consequence of this epidemiological reality, however, is a structural vulnerability in clinical reasoning: when an infectious diagnosis is correct in the overwhelming majority of cases, the cognitive threshold for entertaining an alternative becomes inappropriately elevated. This phenomenon, diagnostic anchoring, in which an early impression resists revision despite discordant subsequent data, has been systematically documented as a leading driver of diagnostic error across specialties and is particularly operative when the initial diagnosis is reinforced by institutional norms and high base-rate pathogens [11]. Both of our patients entered this cognitive trap. Both were receiving anti-TB therapy at the point of referral. Both ultimately required invasive surgical biopsy to establish a cancer diagnosis that, in retrospect, had been present from the outset of their pleural disease.

Study design

The systematic review in the present study was conducted according to the Preferred Reporting Items for Systematic Review and Meta-Analysis (PRISMA) guidelines [12,13].

Search strategy

Literature searches were performed by subject in a number of databases including PubMed, Cochrane, ProQuest, and Wiley. Any discrepancies found were addressed for further discussion among the authors. The search term used in each database is shown in Table 2.

**Table 2:** Keywords for each database.

Database	Keywords	Hits
Pubmed	empyema AND lung cancer	175
Cochrane	empyema AND lung cancer	60
Proquest	("empyema thoracis") AND ("lung cancer" OR malignancy) AND (diagnosis OR misdiagnosis)	43
Wiley	("empyema thoracis") AND ("lung cancer" OR malignancy) AND (diagnosis OR misdiagnosis)	111

Eligibility criteria

The inclusion criteria followed the PICO framework (patient/problem, intervention/exposure, comparison/control, outcome) and comprised (1) type of study: case report from 2006 (20 years); (2) study population: patients presenting with empyema, which was later diagnosed as malignancy; (3) outcomes: diagnostic finding and identification of malignancy. Meanwhile, the exclusion criteria were set to (1) patients with empyema as a complication of already known cancer; (2) irretrievable full-text articles; and (3) non-English articles. Furthermore, duplicate removal was also performed using Microsoft Excel (Microsoft Corp., Redmond, WA, USA). The titles and abstracts of studies were screened according to accessibility criteria by independent reviewers (MFA, YRHS, and SA). Any disagreements were discussed to reach consensus.

Data collection and analysis

Data collection in this study followed the PRISMA flow diagram, including the identification of studies through database searches, screening of duplicates, titles, and abstracts, assessment of full-text articles for eligibility, and extraction and analysis of data from the included studies. We extracted studies manually into the extraction tabulation. The extracted data included the author and year of publication, study location, sample characteristics, number of patients, age, sex, clinical presentation, radiologic findings, laboratory findings, thoracentesis results, procedures performed and their outcomes, cytologic, histopathologic, and other diagnostic findings, treatment, initial diagnosis, and final diagnosis.

Selection of studies

A total of 389 records were identified from electronic databases, including PubMed (n = 175), Cochrane (n = 60), ProQuest (n = 43), and Wiley (n = 111). After removing two duplicate records, 387 records were screened based on titles and abstracts, resulting in the exclusion of 373 records. The remaining 14 reports sought for retrieval underwent a full-text eligibility assessment. Following review, six reports were excluded because the empyema presented as a complication of an already known malignancy, and a history of malignancy was evident. Ultimately, eight studies met the inclusion criteria and were included in the review. The PRISMA flow diagram is shown in Figure 10.

**Figure 10:**
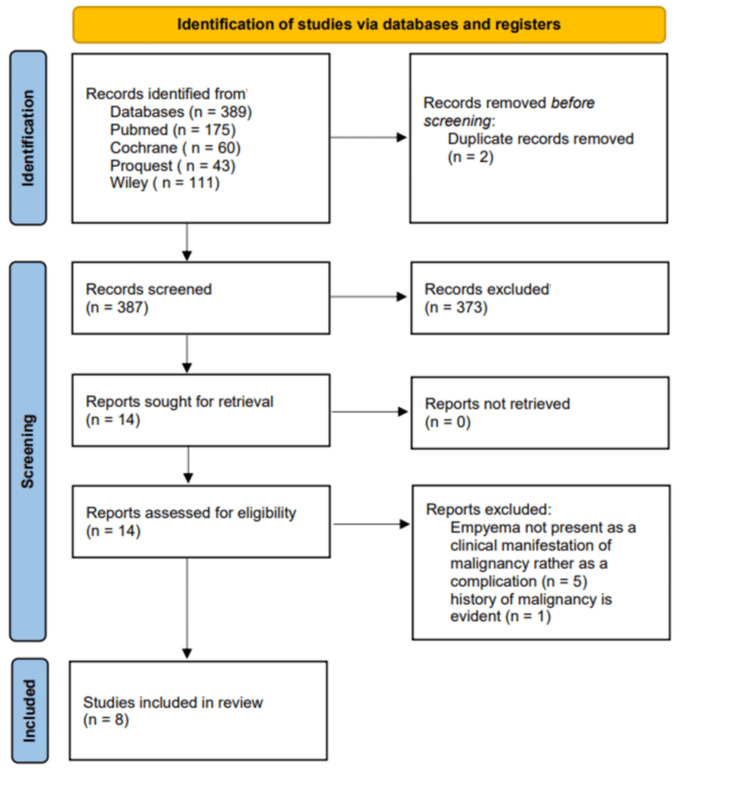
PRISMA flowchart. PRISMA: Preferred Reporting Items for Systematic Review and Meta-Analysis

Characteristics of included studies

All study designs of the included studies were case reports, which were published between 2006 and 2026. The studies were conducted in the following locations: Italy (n = 1), England (n = 1), Egypt (n = 1), Malaysia (n = 1), Portugal (n = 1), Spain (n = 1), Taiwan (n = 1), and Japan (n = 1) [2-9]. The detailed information is shown in Table 3.

**Table 3:** Characteristic studies. SaO_2_: arterial oxygen saturation; RR: respiratory rate; BP: blood pressure; SVC: superior vena cava; Gy: Gray; EGFR: epidermal growth factor receptor; BPH: benign prostatic hyperplasia; PCI: percutaneous coronary intervention; VATS: video-assisted thoracoscopic surgery

Author	Year	Study design	Country	Patient		Presentation	Treatment	Patient outcomes
				Gender	Age			
Leporati A et al. [3]	2020	Case report	Italy	Female	59	1. Dyspnea, vomiting, productive cough with one episode of hemoptysis. 2. Smoked 30 cigarettes for 40 years. 3. Apiretic, with SaO_2_ 89%	Amoxicillin and clavulanic acid	Adenocarcinoma of the lung
Arslan F et al. [4]	2025	Case report	England	Female	55	1. Five-day history of worsening breathlessness and right-sided chest pain. 2. Symptoms began 6 months ago with a productive cough and had streaks of blood. 3. No constitutional symptoms 4. Chronic smoker (one pack per year) 5. RR 35, SaO_2_ 70%	Levofloxacin 500 mg twice daily and metronidazole 500 mg 3x daily	Squamous cell carcinoma in situ → high dose palliative therapy in 13 fractions.
Tseu S et al. [5]	2025	Case report	Egypt	Female	61	1. Productive cough, exertional dyspnea associated with severe pleuritic chest pain 2. Persistent facial and neck swelling over several months 3. Smoker with a 30 pack-year history 4. Phys exam --> right pleural effusion, enlarged lymph node in the right supraclavicular fossa, temperature 38.8, BP 153/88, 1 L oxygen to maintain Sa02 > 94%	Intravenous antibiotics	Small cell lung carcinoma → thoracic radiotherapy (20 Gray (Gy) in five daily fractions over one week) SVC obstruction → dexamethasone 8 mg. After normalization of inflammatory markers, patient started systemic anticancer treatment
Nyanti L et al. [6]	2019	Case report	Malaysia	Male	60	1. Productive cough for 2 months and intermittently blood-stained with febrile 2. Constitutional symptoms of loss weight and appetite. 3. Chronic smoker. 4. Phys exam: dull percussion at the right hemithorax and reduced breath sounds.	Intravenous ampicillin for two weeks, followed by oral ciprofloxacin and ampicillin-sulbactam for four weeks.	Advanced stage adenocarcinoma with wild type EGFR mutation → unable to tolerate chemotherapy and subsequently only received palliative radiotherapy.
Ferreira AI et al. [7]	2022	Case report	Portugal	Male	84	1. Left pleuritic chest pain. 2. 6 months history of productive cough and involuntary weight loss, and one month history of hemoptysis. 3. History of arterial hypertension, dyslipidemia, hypertensive, and ischemic heart disease with reduced ejection fraction, and BPH 4. Afebrile and stable. Pulmonary auscultation of the left hemithorax revealed abolished respiratory sounds in the upper and lower thirds and slightly diminished respiratory sounds in the middle third	Liposomal amphotericin B 3 mg/kg/day	Adenosquamous carcinoma of the lung → progressive pleural effusion despite antifungal therapy, complicated by multidrug-resistant hospital-acquired pneumonia, heart failure, and respiratory failure; the patient died five weeks after hospitalization.
Gonzalez Barcala FJ et al. [8]	2006	Case report	Spain	Male	84	1. 3 days of worsening cough, dyspnea and whitish phlegm. 2. Eupneic; his temperature was 37.0 1C, blood pressure 170/80 mmHg, and heart rate 80 beats/min. 3. Physical examination showed reduced vesicular murmur in the left hemithorax, but no other alterations of note	Thoracentesis	Non-small cell lung carcinoma → Since perforation of a hollow abdominal organ was suspected, urgent surgery was performed, but the patient died during postoperative care.
Matsuoka K [9]	2013	Case report	Japan	Female	75	1. Right chest pain and productive cough 2. History of rheumatoid arthritis for 25 years and ex-smoker for 15 pack-years.	Antibiotics were administered but symptoms did not improve	Sarcomatoid malignant pleural mesothelioma → only supportive care and died five months after diagnosis.
Yao Y and Kuo Y [10]	2023	Case report	Taiwan	Male	68	1. Shortness of breath and chest tightness, particularly during physical activity, for 10 days. 2. Appetite loss --> 4kg weight loss over the past 3 weeks. 3. Past coronary artery disease managed by PCI with one stent implantation and abdominal aortic aneurysm, which was treated by endovascular aneurysm repair 4. Physical exam: breathing sound was decreased in the right side of the chest.	VATS with decortication and delobulation	Malignant pleural mesothelioma with bilateral malignant pleural effusion, epitheloid subtype → cisplatin-based chemotherapy was performed. However, the patient subsequently developed ventilator-associated pneumonia, severe acute respiratory distress syndrome, and septic shock, resulting in death on the 21st postoperative day.

Patient characteristics

We included a total of eight case reports. Based on gender, the occurrence of malignancy presenting as empyema was the same (four males and four females). The age ranged from 55 to 84 years. The history of chronic smoking was reported in five studies. Two studies reported a history of cardiovascular disease, and one study reported a history of rheumatoid arthritis [2-9].

Clinical presentation

Across the reviewed cases, patients commonly presented with non-specific respiratory symptoms, most notably dyspnea and productive cough, often accompanied by pleuritic chest pain. These features are highly consistent with typical presentations of pleural infection, which may contribute to an initial diagnostic bias toward infectious etiologies. In addition to these symptoms, several patients exhibited clinical features suggestive of underlying malignancy, including hemoptysis, unintentional weight loss, and anorexia. Physical examination findings were also largely non-specific, with decreased breath sounds, signs of pleural effusion, and, in some cases, hypoxemia or lymphadenopathy.

Laboratory findings

Laboratory findings were largely consistent with an inflammatory process, frequently demonstrating elevated C-reactive protein levels, leukocytosis, and neutrophilia. However, laboratory parameters were not uniformly consistent, with some patients presenting without significant leukocytosis or only mildly elevated inflammatory markers. In addition, pleural ADA levels, when available, were not indicative of TB, and arterial blood gas analysis showed variable results depending on disease severity.

Certain subtle abnormalities, such as anemia, thrombocytosis, and recurrence of inflammatory markers during the disease course, were observed, which may reflect an underlying chronic or malignant process. Despite these findings, no specific laboratory parameter reliably distinguished malignancy-associated empyema from infectious causes.

Imaging findings

Imaging findings demonstrated a dual pattern that contributed to diagnostic difficulty. Initial radiological evaluation, particularly chest radiography, frequently revealed features consistent with pleural infection, including large pleural effusions, lung collapse, consolidation, or complete opacification of the affected hemithorax. Ultrasound findings further supported this impression, often demonstrating loculated or septated pleural collections typical of empyema.

However, subsequent imaging (CT scan) often revealed features suggestive of underlying malignancy. These included the presence of lung masses, mediastinal lymphadenopathy, bronchial obstruction, and invasion into adjacent structures such as the chest wall or major vessels. In some cases, additional findings such as distant metastases or nodular lesions further supported a malignant etiology. Therefore, persistent or atypical radiological findings, especially the presence of mass lesions, lymphadenopathy, or failure of radiographic resolution, should prompt further investigation for malignancy.

Diagnostic findings

Early diagnostic procedures, including bronchoalveolar lavage, pleural fluid analysis, and cytological examination, often demonstrated findings consistent with infection, such as positive bacterial cultures or inflammatory cell predominance. Notably, pleural fluid cytology was frequently negative for malignant cells, further reinforcing the initial impression of infectious empyema.

Additional investigations, including microbiological studies and TB screening, were commonly performed but often yielded non-specific or negative results. These findings may provide false reassurance and contribute to delays in pursuing further diagnostic evaluation.

Definitive diagnosis was consistently established only after invasive tissue sampling, including bronchoscopic biopsy, pleural biopsy, lymph node biopsy, or surgical procedures such as video-assisted thoracoscopic surgery (VATS) with decortication. Histopathological examination confirmed various types of malignancy, including adenocarcinoma, squamous cell carcinoma, small cell lung carcinoma, and mesothelioma.

Risk of bias

Risk assessment of bias was performed using JBI Case Report appraisal [14]. Overall reporting quality of the included case reports was acceptable. Patient demographics, history, clinical presentation, diagnostic tests, and interventions were consistently well-documented (Q1-Q5, Q8: 100% Yes). However, reporting of post-intervention condition and adverse events was less consistent, with one study rated as no in both Q6 and Q7. The detail of the results of risk of bias was shown in Table 4.

**Table 4:** Risk of bias for each study.

	Leporati A et al. [3]	Arslan F et al. [4]	Tseu S et al. [5]	Nyanti L et al. [6]	Ferreira AI et al. [7]	Barcala FJG et al. [8]	Matsuoka K [9]	Yao Y and Kuo Y [10]
Q1 (Were patient’s demographic characteristics clearly described?)	Yes	Yes	Yes	Yes	Yes	Yes	Yes	Yes
Q2 (Was the patient’s history clearly described and presented as a timeline?)	Yes	Yes	Yes	Yes	Yes	Yes	Yes	Yes
Q3 (Was the current clinical condition of the patient on presentation clearly described?)	Yes	Yes	Yes	Yes	Yes	Yes	Yes	Yes
Q4 (Were diagnostic tests or methods and the results clearly described?)	Yes	Yes	Yes	Yes	Yes	Yes	Yes	Yes
Q5 (Was the intervention(s) or treatment procedure(s) clearly described?)	Yes	Yes	Yes	Yes	Yes	Yes	Yes	Yes
Q6 (Was the post-intervention clinical condition clearly described?)	No	Yes	Yes	Yes	Yes	Yes	Yes	Yes
Q7 (Were adverse events (harms) or unanticipated events identified and described?)	No	Yes	Yes	Yes	Yes	Yes	Yes	Yes
Q8 (Does the case report provide takeaway lessons?)	Yes	Yes	Yes	Yes	Yes	Yes	Yes	Yes

What the systematic review adds is not simply additional cases, but a demonstration of geographic and institutional consistency. Across eight published case reports from Italy, England, Egypt, Malaysia, Portugal, Spain, Japan, and Taiwan [3-10], the same trajectory recurred: a patient with pleural infection, a coherent infectious diagnosis, appropriate therapy, and a failure to improve that was attributed, until tissue sampling intervened, to treatment inadequacy rather than diagnostic error. Histological subtypes varied considerably, encompassing adenocarcinoma, squamous cell carcinoma, small cell lung carcinoma, adenosquamous carcinoma, and both epithelioid and sarcomatoid mesothelioma. This histological diversity is itself analytically important because it establishes that the empyema phenotype is not a peculiarity of any specific tumour biology, but a common downstream consequence of malignant pleural involvement through mechanisms that operate independently of cell type.

A finding of particular mechanistic significance, not previously synthesized in the literature, is that genuine bacterial or fungal co-infection was microbiologically confirmed in the majority of reviewed cases. Isolated organisms included *Klebsiella pneumoniae *and *Staphylococcus aureus*, *Salmonella enterica*, and *Rhodotorula mucilaginosa*, alongside anaerobes and opportunistic fungi documented in related literature [15,16]. This finding reframes the diagnostic problem in a clinically consequential way. Malignancy-associated empyema is not, in most cases, a radiological or clinical mimic of infection, but a genuine infection that is biologically enabled by the malignancy itself. The implications are direct. Antimicrobial therapy is necessary but structurally insufficient, and empyema that resolves with drainage only to recur in the context of ongoing constitutional decline should be interpreted as evidence of a persisting biological driver rather than a failure of infectious management.

Three pathophysiological mechanisms account for the biological enabling of pleural infection by malignancy, and they are not mutually exclusive. The first is endobronchial obstruction with post-obstructive suppuration. Tumour infiltration of a lobar or segmental bronchus produces distal atelectasis, abolishes mucociliary clearance, and converts a region of lung parenchyma into an anaerobic, protein-rich microenvironment that is selectively permissive to bacterial proliferation [17]. This mechanism was most explicitly captured in one report where CT demonstrated right upper lobe bronchial obliteration prior to the isolation of *S. enterica* from pleural fluid, illustrating a causal sequence in which the organism was not the initiating pathology but its consequence. The second mechanism is direct pleural barrier disruption. Tumour invasion of the visceral or parietal pleura alters pleural fluid biochemistry toward a low pH, glucose-depleted, high LDH milieu that constitutes a selective ecological niche for opportunistic organisms [18]. Glucose levels below 1 mmol/L were documented in multiple reports, and the isolation of *R. mucilaginosa*, a fungal pathogen of negligible virulence in immunocompetent hosts, is consistent with this mechanism operating in an advanced pleural microenvironment. The third mechanism is systemic cancer-associated immunosuppression, which lowers the threshold for pleural infection even without structural disruption. This may manifest hematologically as the triad of anemia, thrombocytosis, and disproportionately mild leukocytosis, a pattern consistent with malignancy-related bone marrow infiltration rather than acute sepsis [19].

Superimposed on these mechanisms is a diagnostic failure at the level of pleural fluid analysis that is systematic rather than incidental. Pleural fluid cytology was negative for malignant cells in several reports despite histopathologically confirmed malignancy in each case. This false-negative pattern reflects the dilutional and morphological effects of a neutrophil-dominant inflammatory milieu, which suppresses the relative concentration of shed malignant cells and induces degenerative changes that render them cytologically indistinguishable from reactive mesothelium [20]. The diagnostic literature consistently reports pleural fluid cytology sensitivity for malignancy in the range of 40% to 87%, depending on tumour type and processing method, with mesothelioma generating particularly low yields [21]. In the context of empyema, where inflammatory cell dominance is maximal, this sensitivity is likely to approach its lower bound. A negative cytology result, therefore, must not be interpreted as evidence against malignancy. It is evidence only of the test's structural limitation in this clinical context.

Taken together, the mechanistic and cytological data converge on a single operational conclusion. The clinical fingerprint of malignancy-associated empyema comprises therapy resistance, biochemical incongruence such as low ADA with elevated epithelial tumour markers, co-infection with organisms of low intrinsic virulence, negative pleural cytology, and CT findings that are discordant with a purely infectious etiology. No individual element is pathognomonic. Their co-occurrence, however, constitutes a high-specificity pattern that should systematically override the infectious working diagnosis.

These findings carry direct implications for clinical practice, particularly in resource-limited and TB-endemic settings where the infrastructure for rapid oncological workup may be constrained and where the institutional reflex toward infectious attribution is deeply entrenched. We propose that any patient with empyema who meets two or more of the following criteria should be considered at high risk for underlying malignancy and referred for early invasive tissue sampling without awaiting further evidence of infectious treatment failure. These criteria include age above 50 years with a significant smoking history, failure to demonstrate clinical or radiological improvement after two to four weeks of appropriate antimicrobial or anti-TB therapy, pleural ADA below the TB diagnostic threshold in a TB-endemic context, elevation of serum or pleural CEA, CA-125, or CYFRA 21-1, CT findings demonstrating mass lesion, mediastinal lymphadenopathy, chest wall involvement, or lytic bone lesions, and co-infection with organisms of characteristically low virulence in immunocompetent hosts [18,20]. Diagnostic delay in thoracic malignancy has been independently associated with advanced stage at presentation and reduced overall survival [22], and the cases reviewed here suggest that the empyema phenotype may represent one of its most clinically deceptive contexts.

This report has limitations inherent to its design. Systematic reviews of case reports carry risk of publication bias toward diagnostically atypical or delayed presentations, and the predominantly high-income country origin of reviewed studies limits direct generalizability to the Indonesian clinical context. Prospective cohort data from TB-endemic regions are needed to define the true prevalence of malignancy-associated empyema and to validate the clinical criteria proposed here [23].

## Conclusions

Malignancy presenting as empyema is not a diagnostic curiosity but a coherent clinical syndrome with a reproducible trajectory: infectious attribution, therapy resistance, delayed biopsy, and deferred oncological management. The cases reported herein, situated within a systematic review of eight analogous published reports, demonstrate that this syndrome operates through defined pathophysiological mechanisms, produces genuine co-infection rather than merely simulating it, and is consistently missed by pleural fluid cytology. In TB-endemic settings, where the prior probability of infectious pleural disease is high and anti-TB therapy is initiated empirically, the structural risk of diagnostic anchoring is at its greatest.

The clinical implication is not indiscriminate suspicion, but heightened diagnostic awareness. Therapy-resistant empyema, discordant biochemical findings, and CT features inconsistent with a purely infectious etiology should prompt consideration of underlying malignancy and early tissue diagnosis. Treating the empyema without addressing its oncological substrate will, as both of our cases demonstrate, predictably fail.
